# Simultaneous Detection of Seven *Alternaria* Toxins in Mixed Fruit Puree by Ultra-High-Performance Liquid Chromatography-Tandem Mass Spectrometry Coupled with a Modified QuEChERS

**DOI:** 10.3390/toxins13110808

**Published:** 2021-11-16

**Authors:** Jiali Xing, Zigeng Zhang, Ruihang Zheng, Xiaorong Xu, Lingyan Mao, Jingping Lu, Jian Shen, Xianjun Dai, Zhenfeng Yang

**Affiliations:** 1 Ningbo Academy of Product and Food Quality Inspection (Ningbo Fibre Inspection Institute), Ningbo 315048, China; hellojiali77@gmail.com (J.X.); 1711091100@mail.nbu.edu.cn (X.X.); 1711091102@mail.nbu.edu.cn (L.M.); 1711091103@mail.nbu.edu.cn (J.L.); 1711091104@mail.nbu.edu.cn (J.S.); 2 College of Biological and Environmental Sciences, Zhejiang Wanli University, Ningbo 315100, China; yangzf@zwli.edu.cn; 3 College of Life Sciences, China Jiliang University, Hangzhou 310018, China; p1809085214@cjlu.edu.cn

**Keywords:** *Alternaria* toxins, modified QuEChERS method, ultra-high-performance liquid chromatography-tandem mass spectrometry, mixed fruit puree

## Abstract

The presence of *Alternaria* toxins (*A*Ts) in fruit purees may cause potential harm to the life and health of consumers. As time passes, *A*Ts have become the key detection objects in this kind of food. Based on this, a novel and rapid method was established in this paper for the simultaneous detection of seven *A*TS (tenuazonic acid, alternariol, alternariol monomethyl ether, altenuene, tentoxin, altenusin, and altertoxin I) in mixed fruit purees using ultra-high performance liquid chromatography-tandem mass spectrometry. The sample was prepared using the modified QuEChERS (quick, easy, cheap, effective, rugged, and safe) method to complete the extraction and clean-up steps in one procedure. In this QuEChERS method, sample was extracted with water and acetonitrile (1.5% formic acid), then salted out with NaCl, separated on an ACQUITY UPLC BEH C_18_ with gradient elution by using acetonitrile and 0.1% formic acid aqueous as eluent, and detected by UPLC-MS/MS under positive (ESI^+^) and negative (ESI^−^) electrospray ionization and MRM models. Results showed that the seven *A*Ts exhibited a good linearity in the concentration range of 0.5–200 ng/mL with *R*^2^ > 0.9925, and the limits of detection (LODs) of the instrument were in the range of 0.18–0.53 μg/kg. The average recoveries ranged from 79.5% to 106.7%, with the relative standard deviations (RSDs) no more than 9.78% at spiked levels of 5, 10, and 20 μg/kg for seven *A*Ts. The established method was applied to the determination and analysis of the seven *A*Ts in 80 mixed fruit puree samples. The results showed that *A*Ts were detected in 31 of the 80 samples, and the content of *A*Ts ranged from 1.32 μg/kg to 54.89 μg/kg. Moreover, the content of TeA was the highest in the detected samples (23.32–54.89 μg/kg), while the detection rate of Ten (24/31 samples) was higher than the other *A*Ts. Furthermore, the other five *A*Ts had similar and lower levels of contamination. The method established in this paper is accurate, rapid, simple, sensitive, repeatable, and stable, and can be used for the practical determination of seven *A*Ts in fruit puree or other similar samples. Moreover, this method could provide theory foundation for the establishment of limit standard of *A*Ts and provide a reference for the development of similar detection standard methods in the future.

## 1. Introduction

As a filamentous fungus, *Alternaria* strains are widely distributed in low-temperature and humid environments. *Alternaria* is one of the main microorganisms that cause the decay of fruits and vegetables and other agricultural products during transportation, processing, and storage [1]. As a secondary metabolite secreted by *Alternaria* strains, *Alternaria* toxins (*A*Ts) isolated from *Alternaria* fungi have reached at least 70 compounds. They are commonly divided into five groups according to their great structural divergence [2], namely, (I) dibenzopyranone derivatives of alternariol (AOH), alternariol monomethyl ether (AME), alternene (ALT) and altenusin (ALS) [3]; (II) the tetramic acid derivatives tenuazonic acid (TeA); (III) the perylene derivatives altertoxins (ATX-I, ATX-II, ATX-III); (IV) glycerin tricarboxylic ester compounds (AAL toxin), which can be divided into AAL-TA and AAL-TB; and (V) cyclic tetrapeptides, such as tentoxin (Ten). On the basis of the teratogenic, carcinogenicity, and embryonic toxicity by *A*Ts to human and animals, it is necessary to attach importance to the existence of *A*Ts in food [4].

*A*Ts have been found in various processed products, such as fruits and juices [5], vegetable-based products [6], wheat-based products [7], beer [8], and oil [9]. As a popular processed product, fruit puree is usually made without the addition of any preservatives, flavors, pigments, and other chemicals [10]. Different from other fruit products, fruit puree is another fruit form, while concentrated juice and fruit pulp are artificially manufactured, resulting in loss of nutrients and taste. Therefore, fruit puree is hygienic, nutritious, and healthy, which is suitable as a vitamin supplement for infants, children, and the elderly. Mixed fruit purees contain a certain amount of water, sugar, and other nutrients, which provides suitable conditions for *A*T formation during its processing and storage. The panel of the European Food Safety Authority on contaminants in the food chain evaluated the risks to public health related to *A*Ts in food in 2011 [11] and 2016 [12]. The results in both years showed that infants and young children had the most dietary exposure to *A*Ts. For its unique taste and balanced nutrition, mixed fruit puree has gradually become the mainstream market of infant fruit products [13]. Therefore, special attention must be given to addressing the potential *A*Ts pollution in mixed fruit purees.

The detection of *A*Ts is a necessary step in the safety evaluation of food products contaminated by *A*Ts. The instrumental techniques for measuring *A*Ts mainly include gas chromatography (GC) coupled to a mass spectrometry (MS) detector [14], and liquid chromatography (LC) coupled to an ultraviolet detector [15], a diode array detector [16], enzyme linked immunosorbent assay [17], thin layer chromatography, or a MS detector [18]. Considering that most *A*Ts are stable and unvolatile, GC and GC tandem MS are seldom used for detecting *A*Ts. Nevertheless, recent studies have demonstrated the advantages of the ultra-high-performance liquid chromatography tandem mass spectrometry (UPLC-MS/MS) technique in the determination of *A*Ts in sunflower oil [9], drinking water [19], and wolfberry [20] with high efficiency, precision, and sensitivity [21]. The purpose of the sample pretreatment procedure is usually to extract *A*Ts from food matrices prior to instrumental analysis. Given the complexity of food matrices, sample preparation strategies like solid phase extraction (SPE) or QuEChERS extraction are often required to reach a satisfactory sensitivity [8,22]. Given the complicated SPE operation and the low recovery of some toxins, such as AOH, SPE is unsuitable for the pretreatment of *A*Ts [23]. QuEChERS is a quick, easy, cheap, effective, rugged, and safe sample pretreatment technology based on dispersive SPE and has been successfully used in detecting *A*Ts [24]. The QuEChERS approach has already been applied to 25 mycotoxins in cereals [25] and different kinds of mycotoxins, including the *A*Ts in barley [18] and pomegranate [5]. To date, the QuEChERS method coupled with a UPLC-MS/MS method has successfully been applied to the determination of six *A*Ts (AOH, *A*TS, TeA, AME, ALT, and Ten) in mango [26,27]. However, there is no report on the occurrence of these *A*Ts in mixed fruit puree at present. Considering that simultaneous detection of *A*Ts in mixed fruit puree is very important for infant health, there is an urgent need to establish a QuEChERS method coupled with UPLC-MS/MS to detect the *A*Ts in mixed fruit puree.

A reliable and sensitive detection of *A*Ts was achieved by optimizing the water addition, dehydrating agent, salting out agent, extraction solvent, extraction method, and adsorbent for the QuEChERS procedure. The purpose of this research was to establish a modified QuEChERS method coupled with UPLC-MS/MS for simultaneously determining seven *A*Ts (TeA, AOH, AME, ALT, Ten, ALS, and ATX-I, based on the availability of the standard substance) in mixed fruit purees. Moreover, this method does not require extraction column purification or expensive equipment such as a gel permeation chromatograph, and the pretreatment process is simple. The established method was applied to determine the contents of seven *A*Ts in 80 mixed fruit puree samples.

## 2. Results and Discussion

### 2.1. Optimization of Water Addition

**Figure 1:**
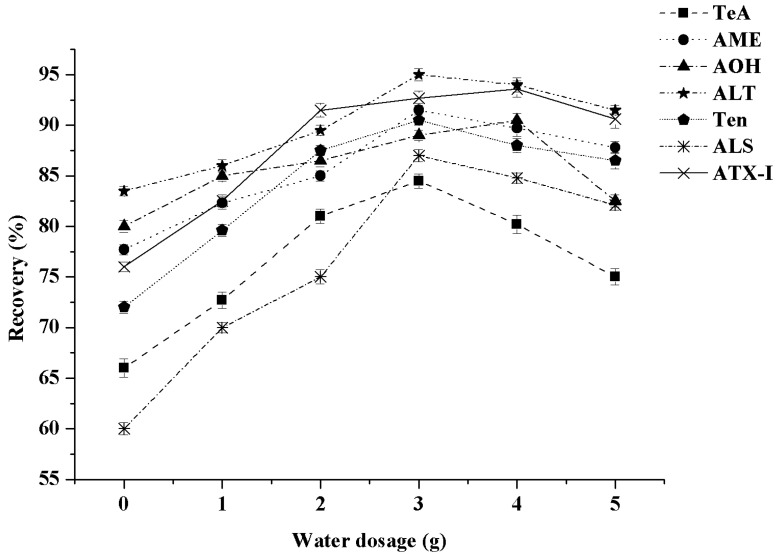
Effect of water content in mixed fruit mud on the recovery of seven kinds of *A*Ts (n = 3).

The rapid detection of pesticide residues in fruits and vegetables through UPLC-MS/MS coupled with QuEChERS has been intensively researched and has revealed that adding a certain amount of water to vegetables and fruits with low water content improves the extraction effects [28]. Considering the high sweetness and viscosity of mixed fruit purees, adding a certain amount of water can increase the recovery rate. In this study, the effects of water dosage (0, 1, 2, 3, 4 and 5 g) on the extraction efficiency were studied. The results (Figure 1) indicated that the best extraction effect, with a recovery rate between 85.1% and 96.4%, was achieved by adding 3 g of water into 5 g of mixed fruit puree. This may be due to the fact that acetonitrile could be better immersed in the sample to improve the extraction effect by adding water [29]. When the amount of water added was more than 3 g, the recoveries of seven *A*Ts was decreased. We deduced that the increased proportion of water would diluted the organic solvents used to extract *A*Ts, resulting in a decreased extraction performance.

### 2.2. Optimization of Extraction Solvent

**Figure 2:**
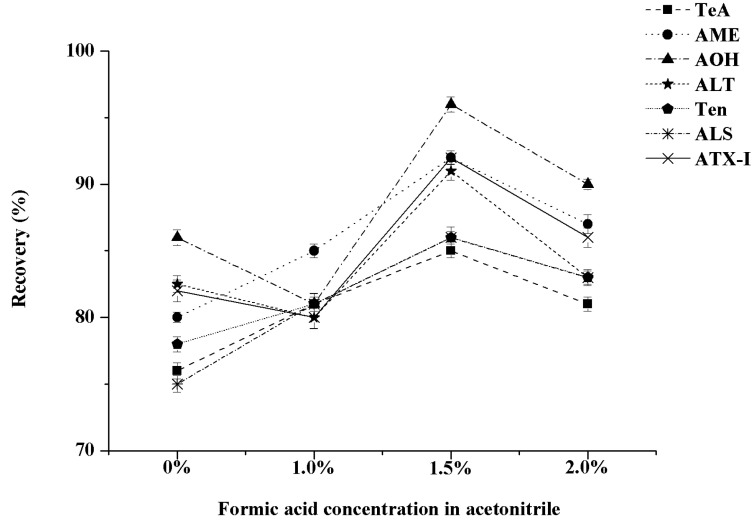
Effect of different FA concentrations on the recovery of seven kinds of *A*Ts in an acetonitrile system (n = 3).

To minimize the interference of the co-extracted materials and improve the extraction efficiency of the seven *A*Ts, the extraction solvent was evaluated. Considering that TeA is highly acidic and more polar than other *A*Ts, it is easy to chelate with metals. Moreover, TeA usually exists in food in the form of salt. Therefore, adding a proper amount of acid to the extraction solvent is conducive to the TeA extraction [30]. In our research, the extraction effects of seven *A*Ts by pure acetonitrile; pure methanol; 1%, 1.5%, 2% FA acetonitrile; and 1%, 1.5%, 2% FA methanol solutions were compared. The results showed that the extraction solution was turbid and the recovery was only approximately 25% when pure methanol and 1%, 1.5%, 2% FA methanol solutions were used as extraction agents, which were much lower than those of the acetonitrile system, although the recoveries of seven *A*TS extracted by pure acetonitrile were low. The recoveries of seven target compounds were between 84% and 96% when the FA content reached 1.5% in acetonitrile, which were higher than those of other extraction solutions. Therefore, 1.5% FA acetonitrile solution was used as the extraction solution for seven *A*Ts. The recovery rates of the seven *A*Ts in the acetonitrile system are shown in Figure 2.

Moreover, the dosage (5, 10, and 15 mL) of 1.5% FA acetonitrile solution on extraction efficiency was also investigated. The results showed that when adding 5 mL of the 1.5% FA acetonitrile solution, the recoveries of the seven *A*Ts were the highest, ranging from 84% to 95%. This result is consistent with the previous studies on the extraction of *A*Ts in fruits and vegetables by De et al. [6] and Dong et al. [31], who used the same extractant and dosage as the optimal extractant to extract *A*Ts. Therefore, 5 mL 1.5% FA acetonitrile solution was selected as the extraction agent in this study.

### 2.3. Optimization of Dehydrating Agent and Salting Out Agent

**Figure 3:**
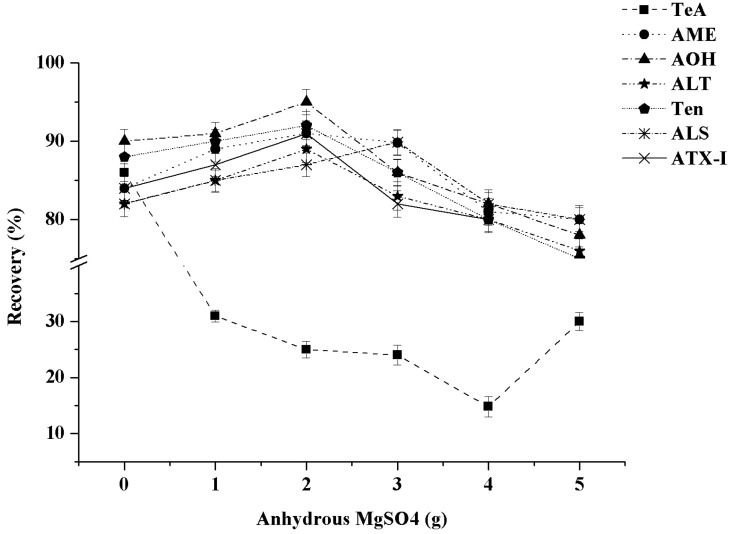
Effect of the dosages of anhydrous MgSO_4_ on the recoveries of seven *A*Ts (n = 3).

Anhydrous MgSO_4_ is usually used to remove the moisture from the sample matrix in the QuEChERS method [32]. The effects of 0, 1, 2, 3, 4, and 5 g of anhydrous MgSO_4_ on the recoveries of the seven *A*Ts were compared in this study. The results indicated that 2 g of anhydrous MgSO_4_ was the optimal dosage of dehydrating agent for the six *A*Ts (Ten, AOH, AME, ALT, ALS, and ATX-I) in the mixed fruit puree samples, while the recovery of TeA was unsatisfactory. As shown in Figure 3, compared with the low recovery of TeA (15–32%) when the anhydrous MgSO_4_ was added, the recovery of TeA was much higher (86%) without anhydrous MgSO_4_. It may be due to the strong chelation of TeA on Mg^2+^, which resulted in a decrease of recovery rate. Meanwhile, the recoveries of the other six *A*Ts had no significant difference whether the anhydrous MgSO_4_ was added or not. Our results were similar to those obtained by Cheng et al. [33] and Chen et al. [34] on the detection of *A*Ts in red jujube and fruits. Thus, anhydrous MgSO_4_ was not used in this study.

**Figure 4:**
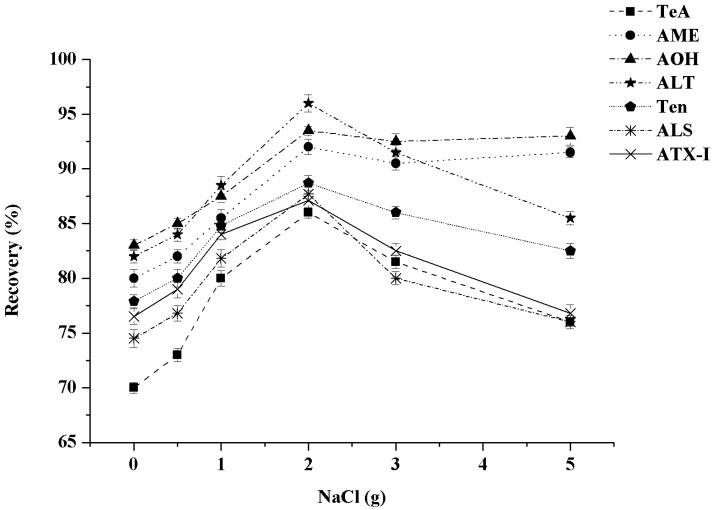
Effect of the dosages of NaCl on the recoveries of seven *A*Ts (n = 3).

With the addition of salting-out agent, the organic phase molecules in the extract will break the hydrogen bond with the water molecules due to the increase in ionic strength, and be salted out from the water, and the extraction efficiency can be greatly improved [35]. The salting out efficiencies of different NaCl dosages (0, 0.5, 1, 2, 3, 5 g) were evaluated in our study. As shown in Figure 4, as the dosage of NaCl increased, the recovery rates of the *A*Ts generally increased first and then decreased. Among them, when the dosage of NaCl was 2 g, the recovery rates of seven *A*Ts were the highest (85.5–96.8%). Therefore, 2 g of NaCl was selected as the salting-out agent in this study.

### 2.4. Optimization of the QuEChERS Purification

The extraction solution showed a deep color after the mixed fruit puree samples were extracted with 1.5% FA in acetonitrile. This may be attributed to impurities, such as natural pigments, which were also extracted into the solution, so the extraction solutions must be purified further to reduce the influence of impurities [36]. QuEChERS purification techniques have been widely applied in the agricultural products and food detection fields [20]. Some adsorbents, such as octadecylsilyl (C18), primary secondary amine (PSA), and graphitized carbon black (GCB), are commonly employed in QuEChERS procedures. In this experiment, the purification effects of different adsorbents and whether to add adsorbent were studied. Since the GCB adsorbent has the same planar structure as the seven *A*Ts, GCB could absorb *A*Ts while absorbing impurities, so GCB was not considered as a purification adsorbent in this study [31]. Then, 2 mL of the upper extraction solvent was accurately transferred into 5 mL centrifuge tube pre-loaded with 50, 100, and 150 mg of C18 and PSA, respectively. The extraction solutions were vortexed and centrifuged. Subsequently, 500 μL of supernatant was mixed with 500 μL of primary water, which was filtered through a 0.22 µm organic filter membrane before detection by UPLC-MS/MS, and then the recoveries were calculated. The upper extractant detection without adsorbent was the same as the above operation.

The purification efficiencies for the seven *A*Ts with the PSA adsorbent were higher than those obtained with C18 (Table 1). Moreover, the purification efficiencies were the highest when the amount of adsorbent was 100 mg. The effect without an adsorbent was similar to that with 100 mg of PSA. In addition, the precision and repeatability of the adsorbent were not ideal with the PSA adsorbent (Table 1). Jiang et al. [32] found that the addition of an adsorbent had no significant effect on the recovery rate of the *A*Ts of citrus, so they did not choose the adsorbent. Besides, Guo et al. [37] found that the effect without any adsorbent was significantly higher than that with any other adsorbent in detecting the *A*Ts of grapes. Finally, no adsorbent was added in the extraction process of *A*Ts in mixed fruit puree.

### 2.5. Optimization of the Extraction Method

**Figure 5:**
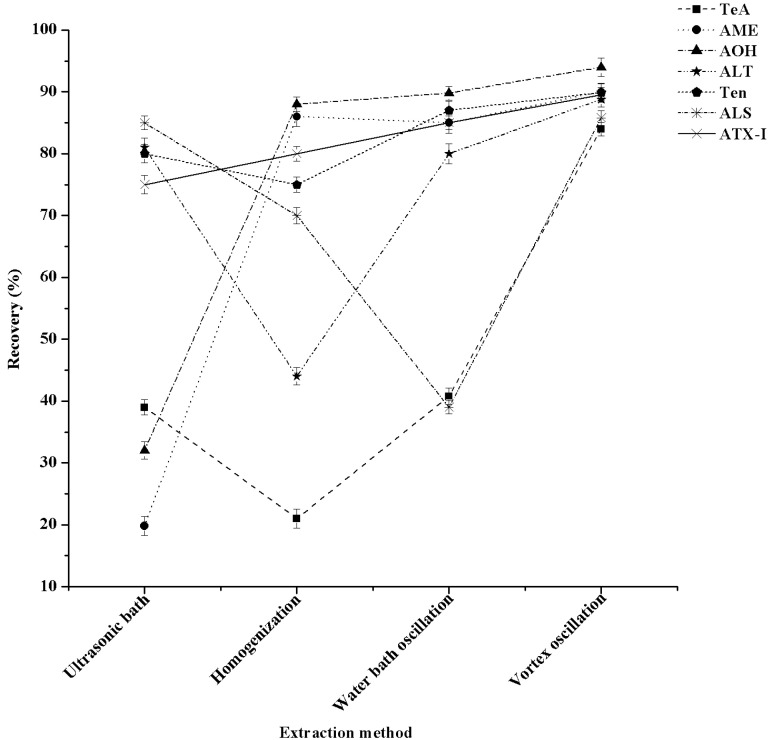
Effect of the four extraction methods on the recoveries of seven kinds of *A*Ts (n = 3).

In this experiment, the efficiency of different extraction methods was studied. They included vortex oscillation (300 r/min, 10 min), homogenization (12,000 r/min, 5 min), ultrasonic bath (40 °C, 20 min), and water bath oscillation (40 °C, 20 min) on the recovery of seven *A*Ts were compared. As shown in Figure 5, when homogenous extraction was used the recoveries of ALT and TeA were 44% and 23%, respectively. We infer that puree sample stuck to the head of the homogenizer during the homogenization extraction process, resulting in excessive substrate loss and severely reducing the extraction effect of some *A*Ts. As the same time, the extractant was not fully contacted with the sample located in the bottom of the centrifuge tube during ultrasonic extraction. Thus, the recoveries of TeA, AOH, and AME were all lower than 40% in ultrasonic extraction. Similarly, under the condition of water bath oscillation, the recoveries of TeA and ALS were also low, at 41% and 38%, respectively. Thus, we could deduce that moderate extractions like water bath oscillations, homogenization, and ultrasonic extraction were not suitable for the extraction of seven *A*Ts. However, the recoveries of the seven *A*Ts in vortex oscillation extraction were significantly higher than those of the other three extraction methods (*p* < 0.05), which exceeded 83%. This may be due to the fact that *A*Ts in the matrix are not easily destroyed during vortex oscillation.

In addition, the effects of vortex oscillation time (5, 10, and 20 min) on the recoveries of the seven *A*Ts were also compared. When the oscillation time was 10 min, the recoveries of seven *A*Ts were the highest (82.1–96.8%). Therefore, 10 min of vortex oscillation was selected as the extraction method in this study.

### 2.6. Optimization of Chromatography and Mass Spectrometry Conditions

Water-methanol and water-acetonitrile are commonly used in UPLC-MS/MS as the mobile phase [38]. Besides, the introduction of FA and ammonium formate can usually enhance the target response and improve the target peak [39]. In our previous study, FA acetonitrile was used as the extractant. To maintain consistency, this experiment focused on three mobile phase systems: water-acetonitrile, 0.1% FA aqueous solution-acetonitrile, and 0.1% FA with 5 mmol ammonium formate solution-acetonitrile. The results showed that the introduction of FA enhanced the response of the seven kinds of target *A*Ts, while the response of the target decreased after the introduction of ammonium formate, and trailing appeared in the peak type. Hence, 0.1% FA aqueous solution-acetonitrile was selected as the mobile phase system.

**Figure 6:**
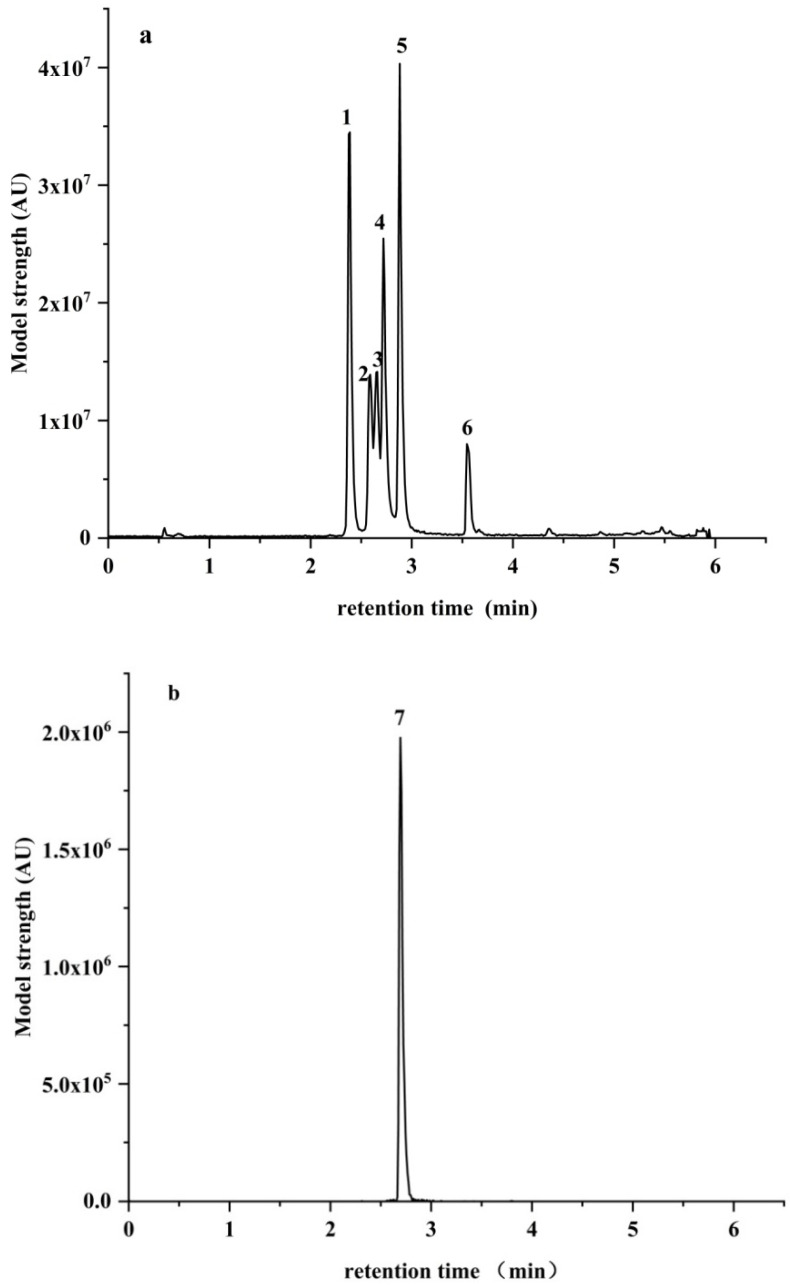
Mass spectrogram of seven *A*Ts under positive (**a**) and negative (**b**) electrospray ionization. Note: 1 for ALT; 2 for ALS; 3 for TeA; 4 for AOH; 5 for Ten; 6 for AME; 7 for ATX-I.

MRM ion mass spectra of the seven kinds of *A*Ts and the total MRM ion mass spectra of ATX-I (negative ions) and other six *A*Ts (positive ions) are shown in Figure 6, respectivly. The qualitative and quantitative ions of each toxin were determined through the continuous injection of the flow injection pump and then optimized by the mass spectrometry conditions (such as conic hole voltage, collision voltage, ion source temperature, desolvent gas temperature and flow, collision gas flow) to achieve the optimal ionization efficiency of each target substance. The samples were respectively scanned using ESI^+^ and ESI^−^ modes to find the parent ion with a high response value. The collision voltage was further changed and secondary mass spectrometry scanning was performed to find the daughter ions with strong signal and stability.

### 2.7. Method Validation

#### 2.7.1. Matrix Effects (MEs)

As with ion enhancement or inhibition, MEs are caused by the influence of co-eluting compounds on the ionization efficiency of the electrospray interface in UPLC-MS/MS analysis [40]. Reportedly, MEs are common in *A*Ts analysis by UPLC-MS/MS [21]. In order to test whether the response value of the blank sample matrix to the target compound was enhanced or inhibited, seven kinds of *A*T mixed solutions were prepared with the solvent standard and matrix blank solution, respectively, at the concentration of 50 ng/mL, and the results were further compared.

As shown in Table 2, the ME values of Ten and ATX-I were between 80% and 100%, which indicated that the MEs of Ten and ATX-I could be ignored. TeA, AOH, and AME showed matrix suppression with ME values lower than 80%. On the contrary, ALT and ALS exhibited matrix enhancement with ME values higher than 120%. All of these indicated that some interfering substances still exist although the extractant solution was purified, which inhibited the analysis of the target analytes remained in the solution. This phenomenon was consistent with the results of other researches [41].

To compensate for the MEs, dilution (5 ng/mL) and a small volume injection (3 μL and 10 μL) were used to quantify the seven *A*Ts in the mixed puree samples. The MEs of AME, Ten, and ATX-I had no significant change (*p* > 0.05) after dilution and injection with 10 μL. However, significantly different MEs (*p* < 0.05) were found for all the seven *A*Ts after dilution and injection with 3 μL. Moreover, the MEs of all the seven *A*Ts were between 80% and 100%. This indicated that both matrix suppression (TeA, AOH, and AME) and matrix enhancement (ALT and ALS) of seven *A*Ts were resolved by dilution and injection with 3 μL (Table 2).

#### 2.7.2. Linearity and Detectability of the Method

In the linearity studies, all the standard working solutions were determined under optimal chromatography and mass spectrometry conditions. Linear regression analysis was performed on a plot with concentration on the X-axis, and the peak area on the Y-axis. The results shown in Table 3 indicate that suitable linearities were obtained in the corresponding concentration range and the coefficients of determination (*R*^2^ values) exceeded 0.990 for all seven *A*Ts.

The LODs and LOQs of the method were calculated according to the validated experimental results. The results showed that the LODs of the seven *A*Ts were in the range of 0.18–0.53 μg/kg, and the LOQs of the seven *A*Ts were in the range of 0.56–2.17 μg/kg (Table 3).

#### 2.7.3. Trueness and Precision of Standard Addition

The trueness and precision of the method were assessed for each toxin by determining the recoveries and the RSDs from the blank mixed fruit puree samples spiked at three different levels (5, 10, and 20 μg/kg). The average recoveries were in the range of 79.5–106.7%, and the RSDs were lower than 9.78% (Table 4). Thus, the trueness and precision of the seven *A*Ts in the mixed fruit purees are acceptable, satisfying the AOAC criteria [42].

#### 2.7.4. Analysis of Fruit Puree Samples

This study established a method for simultaneously determining seven *A*Ts in mixed fruit purees by UPLC-MS/MS coupled with modified QuEChERS.

For the latter procedure, the modified QuEChERS method optimized the water addition, the extraction agent, the dehydrating agent, the salting out agent, the QuEChERS purification, and extraction method to make the pretreatment simpler and more effective. The optimized results showed that the recovery rates of the seven *A*Ts were the highest under the following conditions: 3 mL of primary water was added, 5 mL of 1.5% FA was used as the extraction agent and was extracted by vortex oscillation, no anhydrous MgSO_4_ was used, 2 g of NaCl was used as the salting out agent, and no purifier was added. The established UPLC-MS/MS method can accurately, quickly, and reliably determine *A*Ts. The proposed method has satisfactory applicability and can be used in the risk monitoring of laboratories.

A total of 80 fruit puree samples for infants were determined by the established and validated method. The results showed that the seven *A*Ts were detected in 38.75% (31/80) of the mixed puree samples (Table 5). Besides, the content of TeA was the highest in the detected samples (23.32–54.89 μg/kg) while the detection rate of Ten (24/31 samples) was higher than the other *A*Ts. Furthermore, the other five *A*Ts had similar and lower levels of contamination. For instance, AOH and AME were detected in seven and five samples, which ranged from 3.75 μg/kg to 8.11 μg/kg and 2.28 μg/kg to 9.83 μg/kg, respectively. The samples of ALT, ALS, and ATX-I were detected in one, three, and two cases, respectively. Among them, the content of ALT was 2.66 μg/kg, the contents of ATX-I were 6.43 μg/kg and 7.54 μg/kg, and the contents of ALS were 4.11–15.48 μg/kg. This was an evidence for the contamination of multiple *A*Ts in the mixed fruit puree samples. In general, the concentrations of *A*Ts in mixed fruit puree were higher than those found in cereal-, vegetable-, and (or) fruit-based infant products [43], meaning that they could pose potential health risks to consumers. Thus, monitoring systems should be strictly enforced. In addition, the prevention and control strategies for the pre- and post-processing procedures should be improved.

## 3. Conclusions

In summary, a modified QuEChERS method coupled with a UPLC-MS/MS method was developed and validated for the analysis of seven *A*Ts in mixed fruit puree samples. Under the optimized chromatography and mass spectrometry conditions, mixed fruit puree samples were extracted with 1.5% FA in acetonitrile after adding 3 g water and salting out with 2 g NaCl, without dehydrating and purifying agents. This optimization not only simplifies the procedure, but also improves the recovery rates of the seven *A*Ts. This method had good selectivity, accuracy, and precision when using matrix-matched calibration curves for quantification. The LODs of the method ranged from 0.18 μg/kg to 0.53 μg/kg, and the LOQs were in the range of 0.56–2.17 μg/kg. This method was successfully applied in determining the seven *A*Ts in 80 mixed fruit puree samples. Among all the collected mixed fruit mud samples, 31 samples contained *A*Ts exceeding the levels of LODs, and the content of *A*Ts ranged from 1.32 μg/kg to 54.89 μg/kg. In general, the established method showed good performance in *A*Ts detection with high sensitivity and repeatability, and therefore could be applied to the routine monitoring of *A*Ts in mixed fruit puree. In addition, the newly developed method could effectively identify whether the mixed fruit puree was infected by toxigenic *Alternaria* to ensure the safety of mixed fruit puree and bring economic benefits for the development of the mixed fruit puree industry.

## 4. Materials and Methods

### 4.1. Sample Collection

Different brands of mixed puree samples (80 samples) were collected from different production bases and various supermarkets in Ningbo City, Zhejiang Province, China. The samples were sealed and stored at 4 °C for future use. Mixed fruit puree was mixed and matched by two or three fruits, such as apple, orange, blueberry, kiwi fruit, strawberry, banana, mango, lemon, peach, prune, coconut, pineapple, and blackcurrant.

### 4.2. Chemicals, Reagents, and Standards

Formic acid (FA), acetic acid, and acetonitrile (HPLC-grade) were purchased from Merck Co. (Darmstadt, Germany). Analytical reagent-grade anhydrous magnesium sulfate (MgSO_4_), anhydrous sodium acetate (CH_3_COONa), and sodium chloride (NaCl) were supplied by Sinopharm Chemical Reagent Co., Ltd. (Shanghai, China). Primary secondary amine (PSA) and octadecylsilane (C18), which were used as adsorbents, were all provided by ANPEL Laboratory Technologies (Shanghai) Inc.

Standards of TeA (CAS: 610-88-8), AOH (CAS: 641-38-3), AME (CAS: 26984-49-5), ALT (CAS: 29752-43-0), ALS (CAS: 31186-12-6), ATX-I (CAS: 56258-32-3), and Ten (CAS: 28540-82-1) were all acquired from Anpu Experimental Technology Co., Ltd. (Shanghai, China), and the purities of all the standards exceeded 98%. Each standard substance was prepared by dissolving 1 mg of the amorphous powder in 10 mL acetonitrile to obtain 100 μg/mL standard stock solutions and kept in a refrigerator at −20 °C. The seven individual standard stock solutions were diluted to prepare 1 μg/mL mixed standard solution, which was stored at −4 °C in amber glass vials under darkness before use.

### 4.3. Detection and Quantification Method

The UPLC-MS/MS system used for the separation and quantitation of the seven *A*Ts consisted of a Waters ACQUITY TM UPLC and a Xevo TQ-S mass spectrometer (Waters Technology (Shanghai) Co., Ltd., Shanghai, China). The chromatographic separation was performed on a BEH C18 analytical column (50 mm × 2.1 mm, 1.7 μm, Waters Technology (Shanghai) Co., Ltd., Shanghai, China), and the column temperature was maintained at 40 °C. The flow rate was maintained at 0.4 mL/min, and the injection volume was 3 µL. The mobile phases were water (containing 0.1% FA, *v*/*v*) and acetonitrile. A linear gradient elution procedure was adopted for the separation of the seven *A*Ts. The procedure was as follows: 0–5.0 min, 10–95% (acetonitrile phase); 5.0–7.0 min, 95% (acetonitrile phase); 7.0–7.5 min, 95–10% (acetonitrile phase); and 7.5–10.0 min, 10% (acetonitrile phase).

The mass sepectrometer used a Z-spray electrospray ionization (ESI) source. The ion source parameters were as follows: positive and negative ion switching scanning, capillary voltage of 1.08 kV, source temperature 150 °C, desolvation temperature 600 °C, desolvation gas flow 1000 L/h, and cone gas flow of 150 L/h. The cone voltage (CV), the parent ions, the collision energy (EC), and the fragment ions were optimized for each *A*T using the MassLynx InterlliStar software (Table 6). The seven *A*Ts were analyzed in the multiple reaction monitoring (MRM) mode. Data acquisition and processing were accomplished using the MassLynx ^TM^ 4.2 software.

### 4.4. Sample Pretreatment Method

Sample pretreatment is a key step in sample analysis, as it will affect the accuracy. It includes sample dilution, sample extraction, and sample purification. First, the dosage of the dilution solvent was optimized and the best dosage of dilution solvent was selected by comparing the effects of different water dosages on *A*Ts. Second, the extraction solvent was optimized. With acetonitrile, 1% FA in acetonitrile, 1.5% FA in acetonitrile, 2.0% FA in acetonitrile, methanol, 1% FA in methanol, 1.5% FA in methanol, and 2.0% FA in methanol as the extraction solvents, the best extraction solvent was selected by evaluating the extraction efficiencies with different proportions of these extraction solvents. The effect of the addition of extraction solvent on the extraction efficiency was also evaluated. Finally, the purification process was optimized. With GCB, PSA, and C18 as the adsorbents, the three levels were evaluated. The best adsorbent type and amount were selected by comparing the recoveries obtained with different types and amounts of adsorbent.

In total, 5 g (ME204E, Shanghai Mettler Toledo Instrument Co., Ltd., Shanghai, China) of mixed fruit puree was weighed into a 50 mL plastic centrifuge tube. Then, 3 mL of water, 5 mL of 1.5% FA in acetonitrile, and 2 g of NaCl were added sequentially into the tube. The mixture was vortexed for 10 min (Vortex 3, Guangzhou Yike Laboratory Technology Co., Ltd., ShenZhen, China) and then centrifuged for 5 min at 9500 r/min (TGL-20M, Luxiangyi Centrifuge Instrument Co., Ltd., Shanghai, China). Subsequently, 500 μL of supernatant and 500 μL of water were mixed with the vortex (Vortex 3, Guangzhou Yike Laboratory Technology Co., Ltd., ShenZhen, China) and filtered through a 0.22 µm organic filter membrane. Finally, the supernatant was determined by UPLC-MS/MS.

### 4.5. Method Validation

Exhaustive validation of this newly developed methodology was carried out in terms of the matrix effects (MEs), selectivity, linearity, accuracy (recovery), precision (relative standard deviation).

The MEs were assessed by comparing the peak areas of the mixed matrix standard with those of the mixed solvent standard. The values of the MEs were split into three groups (80–120%, higher than 120% and lower than 80%) based on the determined *A*Ts values. The ME values between 80% and 120% were classified as low MEs, which can be ignored. When the ME values exceeded 120%, they were deemed as matrix enhancements. Meanwhile, the ME values lower than 80% could be classified as matrix suppression. The MEs could be calculated by the following formula [27]:

$$\mathrm{ME}\left(\%\right)=\frac{A_{2}}{A_{1}}\times 100\%$$

In the formula, A_1_ is the average peak area of the toxin standard in pure solvent (initial mobile phase) at a specific concentration, and A_2_ is the average peak area of the toxin standard at the same concentration in the matrix blank solution.

To assess the linearity of the calibration curves, a mixed standard solution of seven *A*Ts was diluted into nine different concentrations (0.5, 1.0, 2.0, 5.0, 10.0, 20.0, 50.0, 100.0, and 200.0 μg/L) using blank mixed fruit puree matrix. The linear equations of the calibration curves were obtained by plotting the concentrations of the seven *A*Ts and the corresponding peak areas, and the correlation coefficients (*R*^2^ values) were calculated. The limits of detection (LODs) and limits of quantification (LOQs) of the seven *A*Ts were determined by serially diluting a mixed standard solution with blank mixed fruit puree matrix solution. The LODs were determined when the signal to noise ratio (S/N) was higher than or equal to 3, and the LOQs were taken when the S/N was higher than or equal to 10.

To evaluate the trueness and precision of the method, mixed standard solution of seven *A*Ts was added to blank mixed fruit puree samples at three different concentrations (5, 10 and 20 μg/kg), and the spiked samples were determined under the optimized pretreatment and analysis conditions. The spiked samples were determined 3 times and the relative standard deviations (RSDs) of the seven *A*Ts were calculated.

## Figures and Tables

**Table 1:** Purification efficiencies of different adsorbent types and amounts for the seven *A*Ts.

Adsorbent	Recovery (%)						
	TeA	AME	AOH	ALT	Ten	ALS	ATX-I
0 mg	84.6 ± 1.2 ^a^	92.1 ± 1.6 ^a^	95.6 ± 2.1 ^a^	90.3 ± 0.9 ^c^	88.2 ± 1.7 ^a^	86.5 ± 2.8 ^b^	91.4 ± 1.7 ^a^
50 mg C18	80.0 ± 3.8 ^d^	86.9 ± 2.9 ^g^	90.1 ± 3.6 ^e^	87.7 ± 4.4 ^f^	80.4 ± 4.6 ^g^	82.6 ± 5.1 ^e^	85.3 ± 3.8 ^g^
100 mg C18	81.4 ± 3.3 ^c^	88.9 ± 4.6 ^e^	92.5 ± 2.9 ^d^	90.3 ± 4.6 ^c^	83.6 ± 3.1 ^d^	84.3 ± 2.9 ^d^	87.7 ± 3.9 ^e^
150 mg C18	75.8 ± 4.0 ^f^	87.2 ± 3.8 ^f^	86.4 ± 3.1 ^g^	88.6 ± 2.9 ^e^	81.9 ± 2.2 ^f^	78.9 ± 3.4 ^g^	86.7 ± 2.2 ^f^
50 mg PSA	80.1 ± 3.1 ^d^	89.7 ± 3.6 ^d^	93.2 ± 2.2 ^c^	90.5 ± 2.8 ^b^	84.4 ± 2.9 ^c^	85.6 ± 3.1 ^c^	89.1 ± 3.2 ^c^
100 mg PSA	83.7 ± 3.9 ^b^	91.6 ± 4.5 ^b^	94.1 ± 5.2 ^b^	91.2 ± 4.3 ^a^	85.9 ± 4.6 ^b^	87.7 ± 2.9 ^a^	90.3 ± 5.6 ^b^
150 mg PSA	77.5 ± 2.8 ^e^	90.2 ± 5.4 ^c^	87.6 ± 4.2 ^f^	89.3 ± 3.9 ^d^	83.4 ± 4.1 ^e^	82.3 ± 3.3 ^f^	88.6 ± 4.1 ^d^

Note: Different letters in the same column represent significant differences (*p* < 0.05).

**Table 2:** Influence of dilution and small volume injection on the MEs.

Target Analyte	Matrix Effects before Dilution (Injection Volume: 10 μL)	Matrix Effects after Dilution (Injection Volume: 10 μL)	Matrix Effects after Dilution (Injection Volume: 3 μL)
TeA	69.7 ± 2.4 ^a^	79.2 ± 1.4 ^b^	85.9 ± 1.9 ^c^
AME	78.6 ± 4.6 ^a^	82.3 ± 3.2 ^a^	88.3 ± 2.1 ^b^
AOH	75.3 ± 3.1 ^a^	87.1 ± 2.8 ^b^	88.6 ± 1.7 ^b^
ALT	123.3 ± 2.5 ^c^	112.4 ± 3.2 ^b^	98.7 ± 0.9 ^a^
Ten	88.4 ± 1.8 ^a^	90.3 ± 2.7 ^ab^	92.2 ± 1.1 ^b^
ALS	136.5 ± 3.4 ^c^	120.5 ± 1.5 ^b^	96.3 ± 1.8 ^a^
ATX-I	90.1 ± 2.0 ^a^	91.7 ± 1.3 ^a^	93.1 ± 2.3 ^b^

Note: Different lines in the same column represent significant differences (*p* < 0.05).

**Table 3:** Linear range, linear equation, *R*^2^, and detection limit of seven kinds of *A*Ts.

Component	Linear Range (ng/mL)	Linear Equation	*R* ^2^	LODs (μg/kg)	LOQs (μg/kg)
TeA	0.5–200	y = 41232.3x − 3133.37	0.9963	0.46	1.47
AME	0.5–200	y = 2828.31x − 893.32	0.9998	0.37	1.22
AOH	0.5–200	y = 2503.73x − 1257.22	0.9997	0.53	2.17
ALT	0.5–200	y = 7573.01x + 248.023	0.9996	0.22	0.77
Ten	0.5–200	y = 16149.8x − 3371.39	0.9998	0.18	0.56
ALS	0.5–200	y = 1398.39x + 618.519	0.9925	0.39	1.25
ATX-I	0.5–200	y = 3136.11x − 1402.31	0.9996	0.27	0.89

**Table 4:** Trueness and precision of the optimized method (n = 3).

Component	Spiked (μg/kg)	Average Recovery (%)	RSD (%)
TeA	5	85.3	9.78
	10	88.2	8.65
	20	79.5	9.65
AME	5	93.0	8.85
	10	93.5	6.54
	20	106.7	5.63
AOH	5	87.2	5.36
	10	96.1	2.35
	20	102.8	7.21
ALT	5	85.6	6.08
	10	90.2	4.68
	20	98.6	6.31
Ten	5	90.3	3.67
	10	88.9	3.69
	20	101.5	5.48
ALS	5	86.0	4.56
	10	86.3	5.13
	20	83.2	5.48
ATX-I	5	91.1	3.43
	10	98.7	2.68
	20	96.5	5.45

**Table 5:** Detection results of the mixed fruit puree samples.

Samples	TeA (μg/kg)	AME (μg/kg)	AOH (μg/kg)	ALT (μg/kg)	Ten (μg/kg)	ALS (μg/kg)	ATX-I (μg/kg)
6	38.92	ND	ND	ND	ND	ND	ND
9	ND	ND	ND	ND	3.26	ND	ND
11	43.31	9.83	7.15	ND	ND	ND	ND
17	ND	ND	ND	ND	5.21	6.56	ND
20	ND	ND	ND	ND	4.73	ND	ND
24	ND	ND	ND	ND	2.11	ND	ND
27	47.96	ND	8.11	ND	4.39	ND	ND
28	ND	ND	ND	ND	5.51	ND	ND
31	ND	ND	ND	ND	2.66	ND	ND
32	52.68	ND	ND	ND	3.67	ND	7.54
35	ND	6.32	7.49	ND	1.66	ND	ND
38	ND	ND	ND	ND	6.32	ND	ND
39	ND	ND	ND	ND	ND	4.11	ND
41	38.99	ND	ND	ND	4.68	ND	ND
43	44.77	ND	ND	ND	8.37	ND	ND
44	ND	ND	4.17	ND	ND	ND	ND
45	ND	ND	ND	2.66	5.18	ND	ND
48	ND	2.28	ND	ND	4.89	ND	ND
50	54.89	ND	ND	ND	2.56	ND	ND
51	43.32	ND	ND	ND	1.69	ND	ND
52	ND	ND	ND	ND	4.67	ND	ND
55	ND	2.61	3.75	ND	4.33	ND	ND
59	ND	ND	ND	ND	5.68	ND	ND
62	34.44	ND	ND	ND	3.65	ND	ND
66	ND	ND	ND	ND	4.66	ND	ND
68	23.32	ND	ND	ND	2.37	ND	ND
70	36.98	ND	5.99	ND	ND	ND	ND
71	ND	ND	ND	ND	1.32	15.48	ND
74	45.67	ND	ND	ND	ND	ND	6.43
76	ND	3.92	4.21	ND	ND	ND	ND
79	33.29	ND	ND	ND	6.98	ND	ND

Note: ND for not detection.

**Table 6:** MS parameters.

Component	Ionization Mode	Parent (*m*/*z*)	Daughter (*m*/*z*)	Dwell Time (s)	Cone Voltage (V)	Collision Voltage (V)
Ten	ESI^+^	415.4	199.2 * 171.2	0.012	25	13 18
AME	ESI^+^	273.2	258.2 128.1 *	0.012	25	25 40
AOH	ESI^+^	259.2	213.2 185.1 *	0.012	25	25 30
TeA	ESI^+^	198.2	125.1 * 153.1	0.012	25	15 12
ALT	ESI^+^	293.2	257.2 * 275.4	0.012	25	12 8
ALS	ESI^+^	291.2	255.2 199.2 *	0.012	25	18 30
ATX-I	ESI^−^	351.3	315.25 * 333.3	0.0.12	25	8 10

Note: * is quantitative ion.

## Data Availability

Not applicable.
